# Longitudinal Lung Function Assessment of Patients Hospitalized With COVID-19 Using ^1^H and ^129^Xe Lung MRI

**DOI:** 10.1016/j.chest.2023.03.024

**Published:** 2023-03-24

**Authors:** Laura C. Saunders, Guilhem J. Collier, Ho-Fung Chan, Paul J.C. Hughes, Laurie J. Smith, J.G.R. Watson, James E. Meiring, Zoë Gabriel, Thomas Newman, Megan Plowright, Phillip Wade, James A. Eaden, Siby Thomas, Scarlett Strickland, Lotta Gustafsson, Jody Bray, Helen Marshall, David A. Capener, Leanne Armstrong, Jennifer Rodgers, Martin Brook, Alberto M. Biancardi, Madhwesha R. Rao, Graham Norquay, Oliver Rodgers, Ryan Munro, James E. Ball, Neil J. Stewart, Allan Lawrie, R. Gisli Jenkins, James T. Grist, Fergus Gleeson, Rolf F. Schulte, Kevin M. Johnson, Frederick J. Wilson, Anthony Cahn, Andrew J. Swift, Smitha Rajaram, Gary H. Mills, Lisa Watson, Paul J. Collini, Rod Lawson, A.A. Roger Thompson, Jim M. Wild

**Affiliations:** a Department of Infection, Immunity and Cardiovascular Disease, University of Sheffield, Sheffield, England; b Sheffield Teaching Hospitals NHS Foundation Trust, Sheffield, England; c National Heart and Lung Institute, Imperial College London, London, England; d Department of Oncology, University of Oxford, Oxford, England; e GE Healthcare, Munich, Germany; f Department of Medical Physics, University of Madison, Madison, WI, USA; g GSK, Stevenage, England; h Department of Radiology, Oxford University Hospitals, Oxford, England; i Oxford Centre for Clinical Magnetic Resonance Research, University of Oxford, Oxford, England; j Department of Physiology, Anatomy, and Genetics, University of Oxford, Oxford, England

**Keywords:** ^129^Xe, COVID-19, gas transfer, hyperpolarized gas, imaging, MRI, xenon MRI

## Abstract

**Background:**

Microvascular abnormalities and impaired gas transfer have been observed in patients with COVID-19. The progression of pulmonary changes in these patients remains unclear.

**Research Question:**

Do patients hospitalized with COVID-19 without evidence of architectural distortion on structural imaging exhibit longitudinal improvements in lung function measured by using ^1^H and ^129^Xe MRI between 6 and 52 weeks following hospitalization?

**Study Design and Methods:**

Patients who were hospitalized with COVID-19 pneumonia underwent a pulmonary ^1^H and ^129^Xe MRI protocol at 6, 12, 25, and 51 weeks following hospital admission in a prospective cohort study between November 2020 and February 2022. The imaging protocol was as follows: ^1^H ultra-short echo time, contrast-enhanced lung perfusion, ^129^Xe ventilation, ^129^Xe diffusion-weighted, and ^129^Xe spectroscopic imaging of gas exchange.

**Results:**

Nine patients were recruited (age 57 ± 14 [median ± interquartile range] years; six of nine patients were male). Patients underwent MRI at 6 (n = 9), 12 (n = 9), 25 (n = 6), and 51 (n = 8) weeks following hospital admission. Patients with signs of interstitial lung damage were excluded. At 6 weeks, patients exhibited impaired ^129^Xe gas transfer (RBC to membrane fraction), but lung microstructure was not increased (apparent diffusion coefficient and mean acinar airway dimensions). Minor ventilation abnormalities present in four patients were largely resolved in the 6- to 25-week period. At 12 weeks, all patients with lung perfusion data (n = 6) showed an increase in both pulmonary blood volume and flow compared with 6 weeks, although this was not statistically significant. At 12 weeks, significant improvements in ^129^Xe gas transfer were observed compared with 6-week examinations; however, ^129^Xe gas transfer remained abnormally low at weeks 12, 25, and 51.

**Interpretation:**

^129^Xe gas transfer was impaired up to 1 year following hospitalization in patients who were hospitalized with COVID-19 pneumonia, without evidence of architectural distortion on structural imaging, whereas lung ventilation was normal at 52 weeks.

FOR EDITORIAL COMMENT, SEE PAGE 572

**Take-home Points**

**Study Question:** Do patients hospitalized due to COVID-19 with no evidence of architectural distortion exhibit longitudinal improvements in ^129^Xe gas transfer to within a normal range between 6 and 52 weeks following hospitalization?

**Results:** At 12 weeks, significant improvements in ^129^Xe gas transfer were observed compared with 6-week examinations. However, ^129^Xe gas transfer remained abnormally low at weeks 12, 25, and 51.

**Interpretation:** In a cohort of patients with moderate severity disease, ^129^Xe gas transfer improved but did not return to within a normal range within 1 year following hospitalization.

In patients hospitalized with pneumonia caused by infection with SARS-CoV-2, the existing literature and clinical experience suggest that there is considerable overlap in clinical presentation with typical pneumonia and ARDS in patients exhibiting hyperinflammation and progressive hypoxemia. However, patients with severe COVID-19 also display evidence of an inflammatory and thrombotic vasculopathy with endothelial dysfunction and excessive blood flow to collapsed lung tissue.^1^, ^2^, ^3^ Abnormal pulmonary vasoregulation has been observed in patients in the acute phase of COVID-19^1^ and may be a pathophysiologic mechanism contributing to the progressive hypoxemia seen in these patients.

Abnormalities on chest radiograph or CT scan imaging at 12 weeks following hospitalization due to COVID-19 are present in some patients, particularly those with more severe disease who require ICU treatment.^4^ However, for patients without radiographic abnormalities, sensitive techniques for monitoring longitudinal change in lung function are needed.

Lung MRI with hyperpolarized ^129^Xe gas allows direct, regionally sensitive measurements of lung ventilation and function, and it is an emerging method that is used both clinically and in clinical research, alongside ^1^H MRI, to evaluate lung function and abnormalities.^5^, ^6^, ^7^, ^8^, ^9^, ^10^, ^11^ In addition, ^129^Xe can image gas diffusion within the lung airspace (diffusion-weighted MRI [DW-MRI]), and the derived apparent diffusion coefficient (ADC) and mean diffusive length scale (Lm_D_) provide three-dimensional in vivo information of the underlying lung microstructure that is highly sensitive to changes in patients with emphysema^12^ and fibrotic lung disease.^13^ In addition, ^129^Xe is soluble in the interstitium/membrane (M) and in the RBCs, and the signal from ^129^Xe in these dissolved compartments can be distinguished spectroscopically. The ratio of the ^129^Xe MRI signal observed in the lung airspaces (gas), the lung M, and bound to the RBCs can thus be determined with magnetic resonance spectroscopic imaging. In particular, the fractions of the ^129^Xe signal in the RBC to M fraction (RBC:M), RBC to gas fraction (RBC:gas), and M to gas fraction (M:gas) ratios have been used to probe gas transfer^14^^,^^15^ and are highly sensitive to gas transfer limitation and longitudinal change in interstitial, emphysematous, and pulmonary vascular diseases.^16^, ^17^, ^18^ RBC:M has been shown to correlate highly with the transfer factor for carbon monoxide (TL_CO_).^6^^,^^19^

Previous studies have reported reduced RBC:M in patients with COVID-19,^20^, ^21^, ^22^, ^23^ including in patients with normal chest CT scan imaging but ongoing dyspnea.^21^ In patients with residual lung abnormalities on CT scans, decreased RBC:M may be due to an increase of xenon uptake in the interstitial lung tissue. However, in the absence of CT scan abnormalities, we propose that a decreased RBC:M instead indicates microvascular (capillary) abnormalities. Therefore, RBC:M in particular may be a sensitive metric suitable for longitudinal assessment of regional gas exchange abnormalities in patients who have had COVID-19 and have normal structural imaging. It is currently unknown whether RBC:M improves longitudinally following COVID-19 pneumonia. Age-related reductions in the RBC:M ratio may be relevant in other cohorts,^24^ and control cohorts well-matched for age are therefore needed for accurate interpretation of RBC:M in post-COVID-19 studies. It is also unclear whether patients with abnormal RBC:M have concurrent abnormalities in lung perfusion or ventilation.

^1^H lung MRI is able to assess changes in lung structure and perfusion. Ultra-short echo time (UTE) imaging enables good visualization of the lung parenchyma and has shown excellent agreement with CT scan imaging in the visualization of lesions in patients with COVID-19.^25^ Dynamic contrast-enhanced (DCE) ^1^H lung MRI allows the assessment of lung perfusion, with high sensitivity and specificity in detecting perfusion defects without exposing the participant to ionizing radiation,^26^ and it is therefore well suited for patient follow-up studies. Increased lung perfusion transit times (time to peak) have been reported in both an acute hospitalized patient with COVID-19 and in nonhospitalized male patients with breathlessness who have had COVID-19.^27^^,^^28^

The current study used a ^1^H and ^129^Xe MRI protocol that combines hyperpolarized ^129^Xe imaging methods sensitive to ventilation, lung microstructure (DW-MRI), and gas exchange (dissolved xenon spectroscopic imaging) alongside ^1^H DCE perfusion and UTE lung structural imaging to assess pathophysiologic changes in patients who had been hospitalized with COVID-19 pneumonia during the postacute period. The primary hypothesis of this work was that abnormal imaging and pulmonary function test (PFT) markers of lung function would increase to within a normal range over the course of 1 year in patients without structural abnormalities seen on CT scan or proton structural imaging. Patients underwent up to four follow-up MRI examinations at approximately 6, 12, 24, and 52 weeks following hospitalization.

## Study Design and Methods

### Participants

Patients with acute COVID-19 pneumonia and no previously diagnosed respiratory disease (excluding mild asthma) were recruited from Sheffield Teaching Hospital's pulmonology and infectious diseases wards from November 2020 to February 2022 for this prospective cohort study, prior to or shortly following discharge. Follow-up ^129^Xe and ^1^H lung MRI examinations were acquired at approximately 6, 12, 24, and 52 weeks following COVID-19 infection. Patients were required to meet the following criteria: (1) a positive SARS-CoV-2 result from a nasal/pharyngeal or respiratory sample; (2) hospitalization with a diagnosis of pneumonia (chest radiograph or CT scan consistent with COVID-19 infection); (3) development of impaired oxygenation (pulse oximetry saturation ≤ 93% on room air) requiring additional oxygen; and (4) no evidence of interstitial lung damage on CT scan or MRI structural imaging at 12 weeks following hospital admission, as judged by a clinical chest radiologist.

Patients with evidence of interstitial lung damage at 12 weeks following hospital admission were recruited into the parallel UK Interstitial Lung Disease Consortium (UKILD) study.^29^ Standard MRI exclusion criteria were applied to all subjects. In addition, patients were excluded if they were unable to tolerate a test inhalation of ^129^Xe gas according to the supervising clinicians’ judgment or if they had a chest size exceeding the ^129^Xe chest coil circumference (76 cm).

Where possible, PFTs were acquired on the same day as the MRI examination at each visit. Spirometry and transfer factor were performed, and from these tests, the metrics FEV_1_, FVC, FEV_1_/FVC, TL_CO_, and carbon monoxide transfer coefficient were calculated and presented as *z* scores and % predicted using Global Lung Function Initiative reference ranges.^30^^,^^31^ This study was approved by the London-Hampstead Research Ethics Committee (REC reference: 9/LO/1115).

### MRI Acquisition

**Figure 1:**
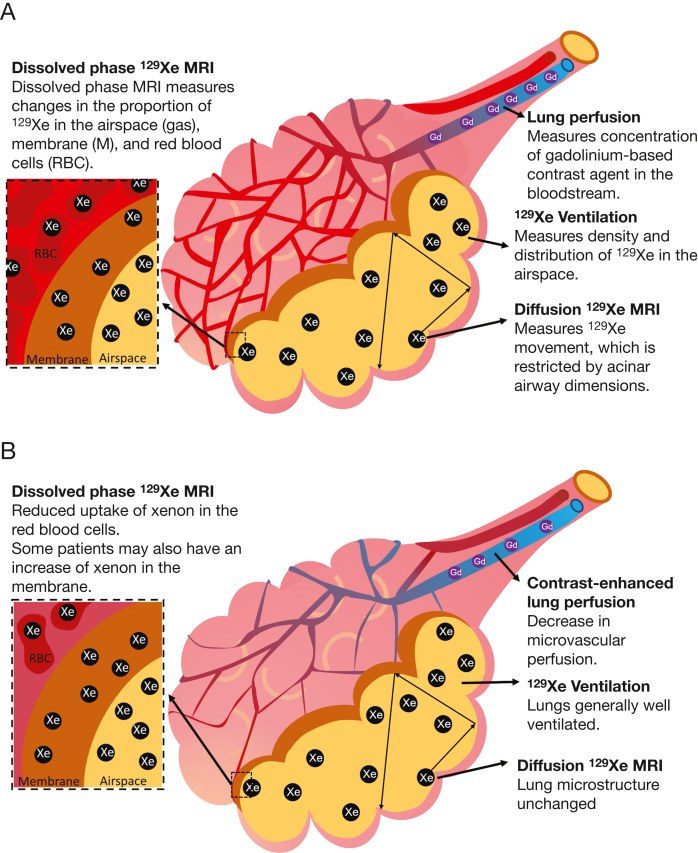
A-B, Illustrative diagram showing how the lung MRI techniques used in this article measure lung perfusion, ventilation, lung microstructure (diffusive length scale), and xenon gas transfer (the transfer of xenon between the airspace, membrane, and RBCs). A, Techniques in a healthy alveolus. B, Possible interpretation of the findings of this article in patients who have had COVID-19, with reduced RBC:M due to damage to pulmonary microcirculation but preserved acinar airway dimensions. RBC:M = RBC to membrane fraction.

Patients underwent scanning on either an HDx 1.5T (N = 7) or a 450w 1.5T (N = 2) (GE Healthcare) MRI scanner.^32^ The ^129^Xe images were acquired with the patient in a flexible quadrature transmit/receive vest coil (Clinical MR Solutions). Patients’ vital signs were monitored throughout the MRI examination. Each patient underwent MRI examinations on the same scanner for baseline visits and follow-up. Figure 1 presents an illustrative diagram of the lung MRI methods used in this study.

^129^Xe doses were polarized to approximately 30% using a home-built high-performance spin-exchange optical pumping polarizer.^32^ This had regulatory approval for manufacture of hyperpolarized ^129^Xe for clinical lung MRI by the UK Medicines and Healthcare Products Regulatory Agency.

MR imaging was conducted as summarized in the following text (parameters are detailed in e-Table 1).

A structural ^1^H scan was acquired following inhalation of a bag of air to match the lung inflation state of the subsequent xenon sequences. ^129^Xe ventilation images were acquired using a three-dimensional imaging sequence with whole-lung coverage following inhalation of a 1 L maximum mixture of ^129^Xe and nitrogen (titrated if subject height < 160 cm^33^) and inhaled from functional residual capacity; patients were coached in the required breathing maneuver prior to their MRI examination.^34^

^129^Xe DW-MRI to assess alveolar microstructural change was acquired following inhalation of a maximum 1 L mixture of ^129^Xe and nitrogen (three-dimensional spoiled gradient echo [SPGR] multiple b-value sequence with compressed sensing with whole-lung coverage).^35^

Three-dimensional spectroscopic imaging of the gas and dissolved phase xenon resonances (dissolved xenon in lung M and in blood RBCs) was acquired by using a maximum 1 L dose of hyperpolarized ^129^Xe (repetition time = 15 milliseconds, flip angle = 22° [three-dimensional acquisition with whole-lung coverage]).^6^

^1^H MRI was acquired by using an eight-element cardiac array (GE Healthcare). UTE images were acquired with a three-dimensional radial sequence during 8 min of free-breathing with prospective respiratory bellows gating on expiration.^36^

Three-dimensional variable flip angle SPGR images^37^^,^^38^ were acquired (flip angle = 2°, 4°, 10°, and 30°) to allow for the correction of lung T_1_ and proton density. DCE lung perfusion MRI was acquired (three-dimensional volumetric time-resolved SPGR acquisition). A half dose (0.05 mL/kg) of Gadovist (Bayer) was administered at an injection rate of 4 mL/s followed by a 20 mL saline flush at 4 mL/s. Patients were advised to hold their breath for as long as possible and breathe shallowly thereafter.

### Image Analysis

Qualitative assessments of the UTE ^1^H structural, ^129^Xe ventilation, and DCE lung perfusion images were made by two radiologists with 10 and 14 years of experience, respectively. UTE images were assessed for parenchymal changes, and ventilation and perfusion images were assessed for defects.

Metrics of ventilation defect percentage (VDP), low ventilation percentage (VP), normal VP, and hyper VP for each patient were calculated by using linear binning (see the Methods section of e-Appendix 1). The coefficient of variation of the segmented lung ventilation images was also calculated from the ^129^Xe ventilation images as a marker of ventilation heterogeneity.

Maps of ^129^Xe ADC and Lm_D_ from a stretched exponential model of ^129^Xe gas diffusion in the lungs were calculated on a voxel-by-voxel basis.^39^

Maps of gas transfer ratios (RBC:M, RBC:gas, and M:gas) were calculated from three-dimensional spectroscopic imaging. The transverse relaxation time (T_2_∗) of the RBC and M spectroscopic peaks was also calculated.

Mean values of all global metrics were calculated for each patient. A sample size calculation was not performed because this was an exploratory study.

Global MRI metrics from visits 1, 2, 3, and 4 were compared by using a Skillings-Mack test due to the presence of missing data^40^ with pairwise Wilcoxon tests and a correction for multiple testing,^41^ implemented by using R software.^42^ Data are presented as median (range), unless otherwise stated.

Mixed linear effect models were set up using a random intercept model to test the relationship between RBC:M and the following: (1) pulmonary blood volume; (2) pulmonary blood flow; (3) mean transit time; (4) VDP; and (5) TL_CO_ *z* score. IBM SPSS Statistics 27 (IBM SPSS Statistics, IBM Corporation) was used for analysis. A *P* value < .05 was considered statistically significant.

### Age- and Sex-Matched Healthy Volunteer Metrics

Median ADC and Lm_D_ values for an age- and sex-matched control cohort were determined by retrospective analysis of previously published data.^43^ Eleven subjects from this previously published work were selected based on matching median and interquartile range (IQR) of age and sex ratio from a cohort of 23 subjects while blinded to MRI metrics; the control cohort had a median age of 63 (40-70) years, and 73% were male.

Median RBC:M, RBC:gas, and M:gas for an age- and sex-matched control cohort were determined by retrospective analysis of a healthy cohort data set, with control subjects chosen based on matching median and IQR of age and sex ratio while blinded to MRI metrics. Twelve subjects were selected (median age, 57 [41-68] years), and 67% were male.

## Results

**Figure 2:**
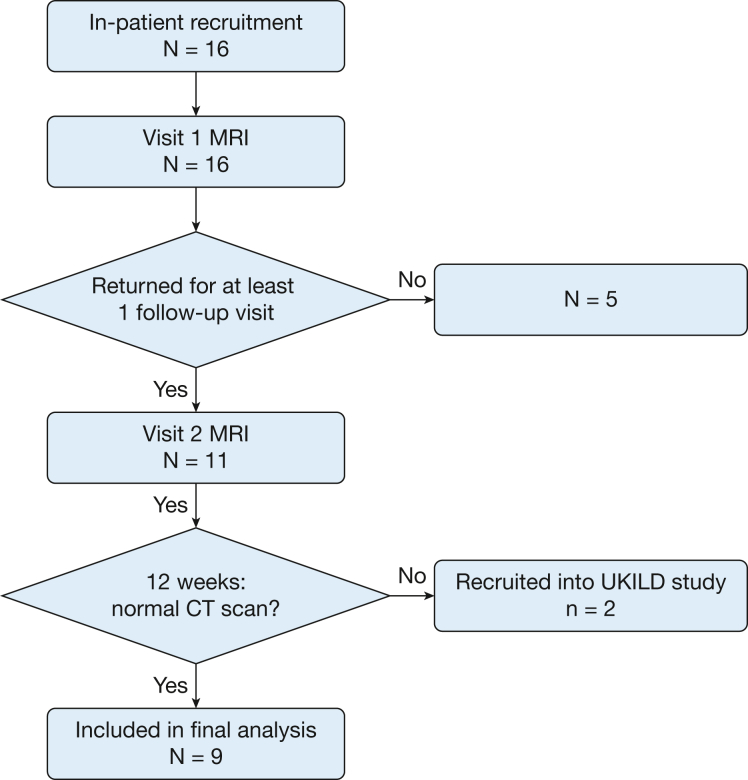
Flow chart of patient recruitment. UKILD = UK Interstitial Lung Disease Long-COVID-19 study.

Of the 16 recruited patients, 14 showed no signs of interstitial lung damage at 12 weeks and were therefore included as part of this study. Nine of 14 patients had follow-up examinations and were included for analysis (Fig 2).

Six of nine patients were male. Median patient age, height, and weight were 57 (42-72) years, 173 (170-191) cm, and 101 (84-112) kg, respectively. Visit 1 (N = 9) occurred 6 (4-12) weeks following hospital admission; visit 2 (N = 9) occurred 12 (11-22) weeks following hospital admission; visit 3 (n = 7) occurred 25 (23-28) weeks following hospital admission; and visit 4 (n = 8) occurred 51 (49-62) weeks following hospital admission. Patients had been admitted to the hospital with COVID-19 for 6 (2-15) days. Further patient demographic data are presented in Table 1.^44^^,^^45^ No patients received any trial of pharmacologic treatment for post-COVID-19 symptoms following discharge. Two of the patients commenced treatment for diabetes during the follow-up period.

**Table 1:** Patient Demographic Data

Characteristic	Group	No. of Patients
Demographic characteristics		
Age, y	< 50	2
	50-59	3
	60-69	3
	70-79	1
Sex	Male	6
	Female	3
BMI, kg/m^2^	25-29.9	3
	30-39.9	5
	≥ 40	1
Comorbidities 4C score^44^	0	4
	1	4
	3	1
Tobacco use history	No tobacco use	4
	Former tobacco use	5
Clinical characteristics on admission		
Admission SF ratio	< 200	0
	200-299	3
	300-399	3
	≥ 400	3
Clinical characteristics during admission		
Maximum oxygen requirement during hospital stay	< 28%	1
	28%-35%	4
	40%	2
	> 60%	2
	CPAP	1
ISARIC 4C score^44^	1-4	3
	5-8	5
	> 8	1
Length of stay, d	1-5	4
	6-9	4
	> 10	1
Maximum National Early Warning Score 2 score^45^	5-6	4
	≥ 7	5
Medication during stay	Oral antibiotics	1
	IV antibiotics	2
	Dexamethasone	9
	Remdesivir (Gilead Sciences)	5
	Immunomodulation therapy	3
	Convalescent plasma	2
	Colchicine	2
	Aspirin	3
	Included in an interventional study?	7
Lowest SF ratio during stay	< 200	2
	200-299	3
	300-399	4
	≥ 400	0
Maximum Fio_2_ during stay	< 28%	1
	28%-35%	4
	40%	2
	> 60%	2
	CPAP	1
Clinical characteristics postdischarge		
MRC Dyspnoea Scale (1-5), 6 wk	1	6
	2	2
	Not available	1
MRC Dyspnoea Scale (1-5), 3 mo	1	7
	2	1
	3	1
	Not available	0
Readmittance	Yes	1 for general surgery unrelated to COVID-19
	No	8

Individuals who formerly used tobacco use all reported ≤ 15 pack y. The pulse oximetry saturation/Fio_2_ ratio (SF ratio) was calculated by using estimated Fio_2_ based on flow rate when delivered by nasal cannulae. ISARIC = International Severe Acute Respiratory and Emerging Infection Consortium; MRC = Medical Research Council; pulse oximetry saturation/Fio_2_ (SF).

UTE and ^129^Xe MRI were successfully acquired in all patients at all visits. DCE lung perfusion imaging was successfully acquired in six of nine patients at visit 1, eight of nine patients at visit 2, six of seven patients at visit 3, and five of eight patients at visit 4. The reasons for unsuccessful lung perfusion imaging were patients failing screening (6 visits), patient motion (2 visits), and technical issues (1 visit).

**Figure 3:**
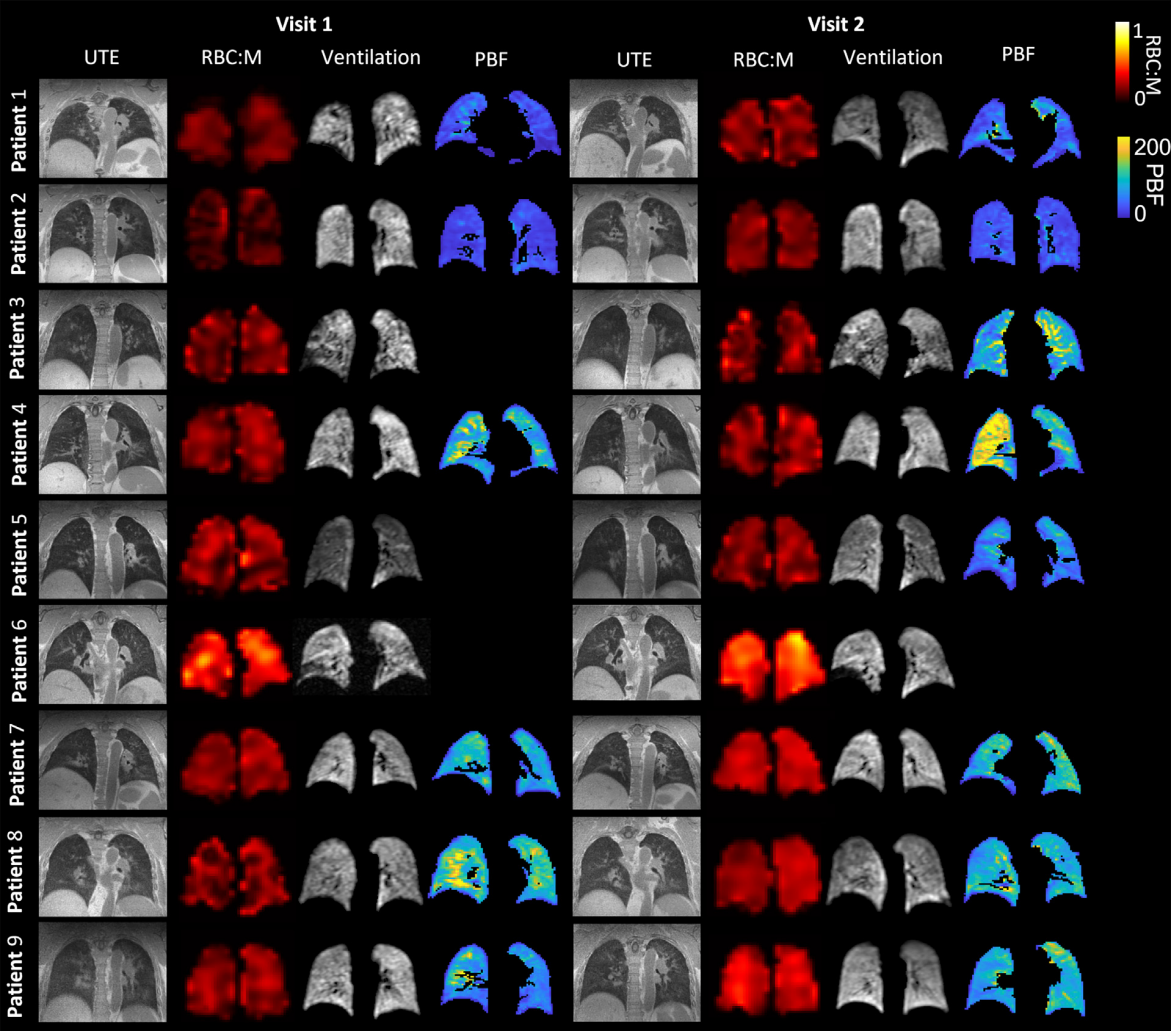
Example of UTE images, RBC:M maps, ^129^Xe ventilation images, and maps of pulmonary blood flow at visit 1 and visit 2, for each patient. The white arrow indicates a segmental perfusion defect visible at visit 1, which improves at visit 2. M = membrane; PBF = pulmonary blood flow; RBC:M = RBC to membrane fraction; UTE = ultra-short echo time.

**Figure 4:**
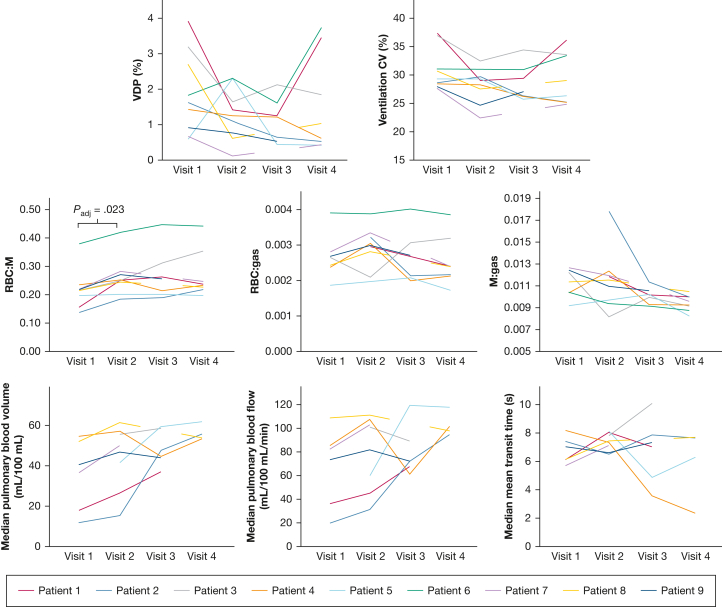
Spaghetti plots of ventilation, dissolved phase xenon, and dynamic contrast-enhanced lung perfusion metrics at visits 1 to 4. CV = coefficient of variation of lung ventilation; M = membrane; M:gas = membrane to gas fraction; RBC:gas = RBC to gas fraction; RBC:M = RBC to membrane fraction; VDP = ventilation defect percentage.

Figure 3 shows representative slices from the UTE images, RBC:M maps, ^129^Xe ventilation images, and DCE pulmonary blood flow maps for each patient at visit 1 and visit 2. Figure 4 displays plots of ventilation, dissolved phase ^129^Xe, and DCE lung perfusion metrics for each patient at each visit. Median metrics and statistical comparisons of metrics at each visit are presented in Table 2.

**Table 2:** Median Metrics for All MRI Parameters at Visits 1, 2, 3, and 4

MRI Parameter	Visit 1	Visit 2	Visit 3	Visit 4	*P* Value	Adjusted *P* Value
No.	9	9	7	8		
ADC, cm^2^/s	0.0344 (0.309-0.0373)	0.0327 (0.0281-0.0386)	0.0340 (0.310-0.364)	0.0338 (0.307-0.0357)	…	…
Lm_D_, μm	281 (260-300)	273 (251-301)	278 (263-290)	279 (263-288)	…	…
RBC:M	0.22 (0.15-0.37)	0.25 (0.18-0.41)	0.25 (0.19-0.44)	0.23 (0.19-0.44)	V1-V2, *P =* .004 V1-V3, *P =* .047 V1-V4, *P =* .039	V1-V2, *P =* .023 V1-V3, *P =* .094 V1, V4, *P =* .094
RBC:gas	0.0026 (0.0018-0.0039)	0.0030 (0.0019-0.0038)	0.0026 (0.0020-0.0040)	0.0024 (0.0017-0.0038)	…	…
M:gas	0.0113 (0.0091-0.0125)	0.0114 (0.0081-0.0179)	0.0101 (0.0091-0.0113)	0.0094 (0.0082-0.0104)	V1-V4, *P =* .031 V2-V4, *P =* .023 V3-V4, *P =* .031	V1-V4, *P =* .063 V2-V4, *P =* .063 V3-V4, *P =* .063
M T_2_∗, ms	2.58 (2.46-2.68)	2.47 (2.38-2.58)	2.42 (2.36-2.56)	2.22 (1.94-2.40)	V1-V2, *P =* .044 V1-V3, *P =* .023 V1-V4, *P =* .008 V2-V4, *P =* .008 V3-V4, *P =* .031	V1-V2, *P =* .053 V1-V3, *P =* .031 V1-V4, *P =* .023 V2-V4, *P =* .023 V3-V4, *P =* .047
RBC T_2_∗, ms	2.20 (2.05-2.48)	2.16 (2.01-2.49)	2.16 (2.06-2.32)	2.27 (2.10-2.47)	…	…
DCE PBV, mL/100 mL	37.8 (11.7-53.5)	47.6 (15.0-60.2)	45.3 (36.3-58.4)	53.8 (52.46-60.72)	…	…
SD PBV, mL/100 mL	18.0 (7.5-28.5)	21.3 (13.0-24.8)	23.8 (17.9-25.7)	21.1 (20.8-22.0)	…	…
IQR PBV, mL/100 mL	25.1 (7.8-34.9)	27.8 (10.5-36.1)	35.9 (24.2-41.5)	29.5 (27.0-31.9)	…	…
DCE PBF, mL/100 mL/min	76.9 (19.6-107.2)	90.2 (30.7-109.5)	71.1 (60.3-117.7)	98.3 (93.4-116.2)	…	…
SD PBF, mL/100 mL/min	45.8 (11.5-58.8)	54.5 (32.0-75.0)	48.6 (35.6-69.5)	54.2 (41.0-65.4)	…	…
IQR PBF, mL/100 mL/min	54.0 (14.1-61.3)	59.2 (25.5-102.9)	59.0 (43.2-78.7)	64.8 (54.2-72.4)	…	…
Median MTT, s	6.5 (5.6-8.0)	7.3 (6.4-8.0)	7.1 (3.5-9.9)	6.9 (2.3 – 7.6)	…	…
SD MTT, s	1.3 (0.9-1.6)	1.2 (0.6-2.6)	1.3 (0.5-2.2)	0.7 (0.6-0.9)	…	…
IQR MTT, s	1.3 (1.1-2.0)	1.6 (0.7-3.1)	1.3 (0.6-3.5)	0.7 (0.5-1.1)	…	…
VDP, %	1.6 (0.6-3.9)	1.3 (0.7-2.6)	1.2 (0.4-2.1)	0.8 (0.4-3.7)	V1-V3, *P =* .016	V1-V3, *P =* .094
Normal VP, %	76.4 (62.5-77.7)	76.9 (72.3-86.2)	78.9 (67.4-81.5)	81.1 (69.0-82.6)	V1-V2, *P =* .027 V1-V3, *P =* .031	V1-V2, *P =* .093 V1-V3, *P =* .093
Low VP, %	12.5 (10.0-15.4)	11.9 (8.9-13.1)	10.9 (9.1-14.8)	10.6 (10.0-13.7)	…	…
Hyper VP, %	11.7 (9.5-18.3)	11.0 (4.2-13.3)	9.7 (7.9-15.8)	8.4 (6.8-15.2)	…	…
Lung ventilation CV, %	29.0 (27.5-37.1)	28.8 (22.3-32.2)	26.9 (25.6-34.1)	26.1 (25.0 -33.3)	V1-V2, *P =* .040 V1-V3, *P =* .016 V1-V4, *P =* .040	V1-V2, *P =* .078 V1-V3, *P =* .078 V1-V4, *P =* .078

Data are presented as median (range) of all patients with available data for each visit. If a Skillings-Mack test determined that there was a significant difference between at least two variables, *P* values are shown for Wilcoxon pairwise tests. *P* values are shown prior to and following adjustment for multiple testing. ADC = apparent diffusion coefficient; CV = coefficient of variation; DCE = dynamic contrast enhanced; IQR = interquartile range; Lm_D_ = mean diffusive length scale; M = membrane; M:gas = membrane to gas fraction; MTT = mean transit time; PBF = pulmonary blood flow; PBV = pulmonary blood volume; RBC:gas = RBC to gas fraction; RBC:M = RBC to membrane fraction; T_2_∗ = transverse relaxation time; V = visit; VDP = ventilation defect percentage; VP = ventilation percentage.

### ^129^Xe MRI

#### Ventilation

**e-Fig 1:**
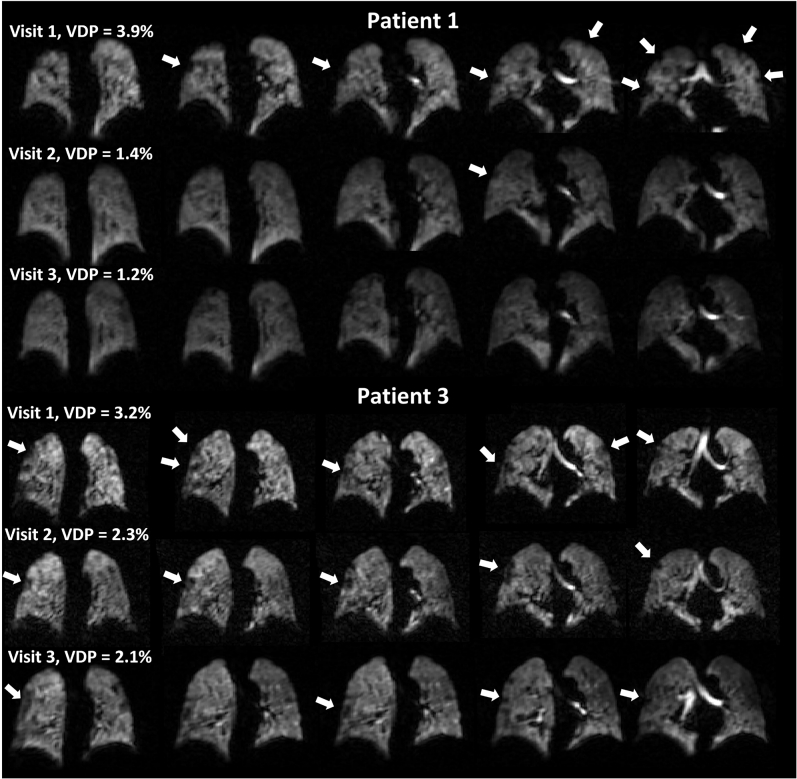


At visit 1, small ventilation defects were visible in the lung periphery in four patients (1, 3, 4, and 6). No other patients had visible lung ventilation defects. At visits 2 and 3, the ventilation defects observed in patients 1, 3, 4, and 6 had improved, with small defects still visible, particularly in patient 3. At visit 4, small peripheral ventilation defects were observed in patients 1, 3, and 6 (e-Fig 1, Fig 3).

Whole lung VDP was calculated for each patient. At visit 1, median VDP was 1.6% (0.6%-3.9%); at visit 2, VDP was 1.3% (0.7%-2.6%); at visit 3, VDP was 1.2% (0.4%-2.1%); and at visit 4, VDP was 0.8 % (0.4%-3.7%).

Quantitative metrics of ventilation improved at visits 2, 3, and 4 compared with visit 1; however, this was not statistically significant following adjustment for multiple corrections (Table 2).

#### DW-MRI (Alveolar Microstructure)

**e-Fig 2:**
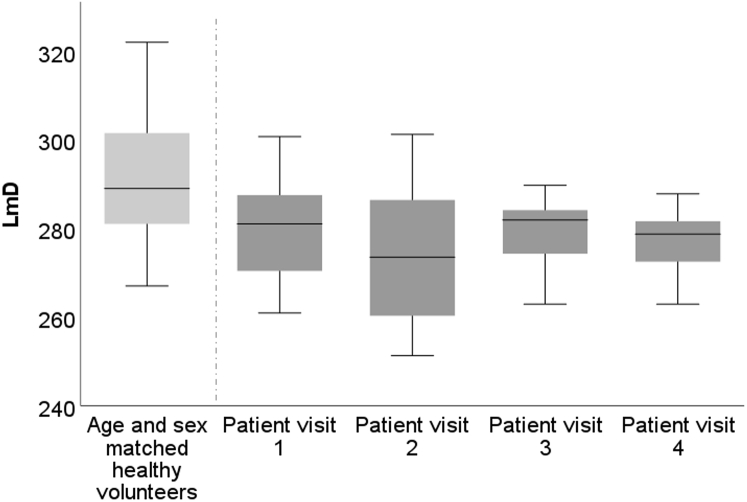


Median ADC and Lm_D_ at each visit are reported in Table 2. No significant longitudinal changes in ADC and Lm_D_ were seen between visits. Median ADC and Lm_D_ were within the median ± IQR of age- and sex-matched control data (age- and sex-matched control data: median ADC, 0.0360 cm^2^/s [IQR, 0.005 cm^2^/s]; median Lm_D_, 289 μm [IQR, 27 μm]) at all visits (e-Fig 2).

#### Dissolved Xenon (Gas Exchange)

**Figure 5:**
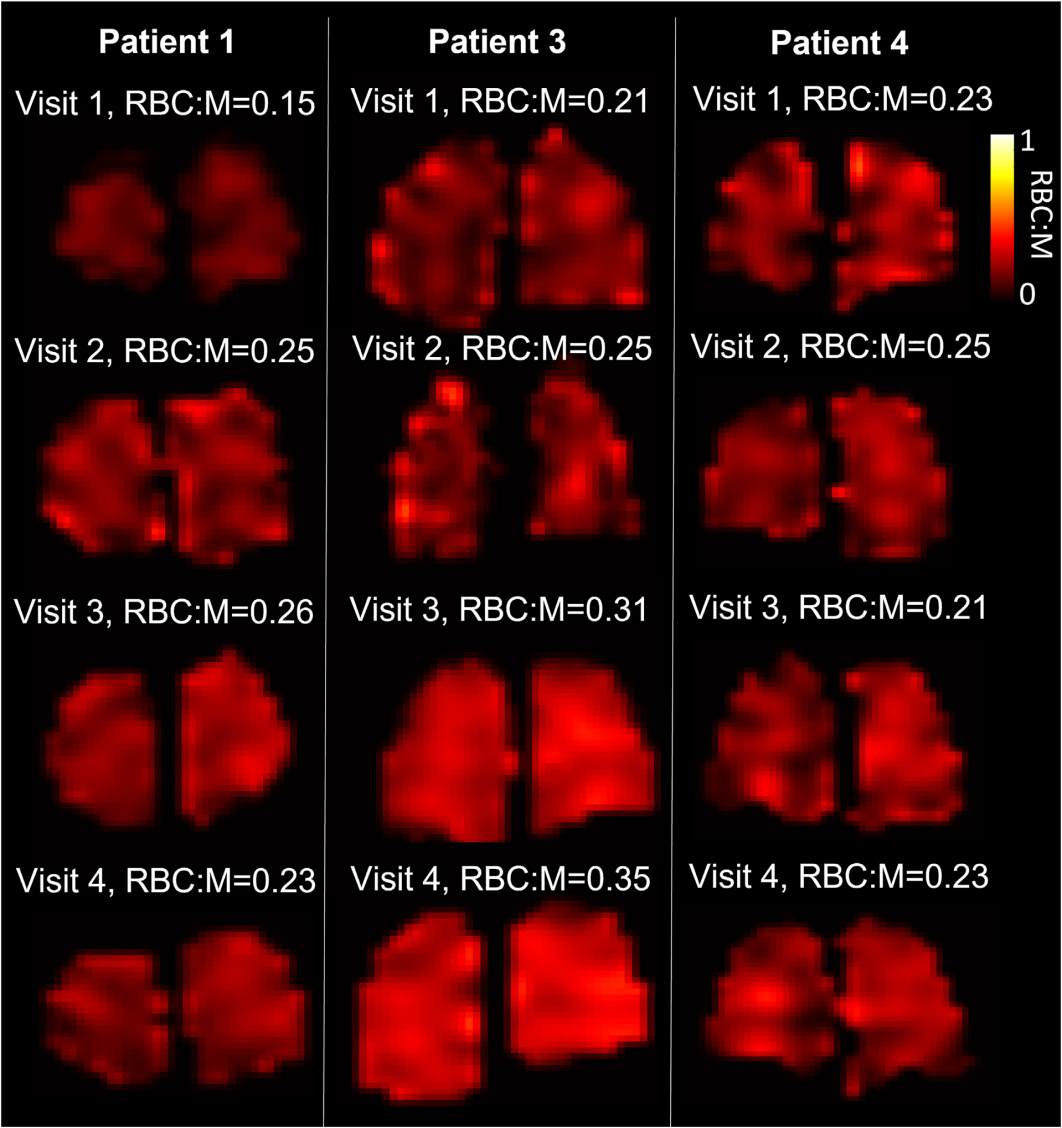
Lung RBC:M maps in three patients with four MRI visits at 6, 12, 25, and 51 weeks following hospital admission. Mean RBC:M at each visit is shown. M = membrane; RBC:M = RBC to membrane fraction.

Figure 5 presents sample RBC:M maps. The global RBC:M ratio significantly increased at visit 2 compared with visit 1 (*P*_adj_ = .023). RBC:M at visit 1 was 0.22 (0.15-0.37), and at visit 2 it was 0.25 (0.18-0.41). No subjects showed a decrease in RBC:M at visit 2 compared with visit 1 (Figs 4, 5). RBC:M at visits 3 and 4 were 0.25 (0.19-0.44) and 0.23 (0.19-0.44), respectively. At visits 3 and 4, some patients showed continued improvement (Figs 3, 4), while others maintained an abnormal RBC:M during the 25- to 51-week period. There were no significant changes between visits 2, 3, and 4.

**Figure 6:**
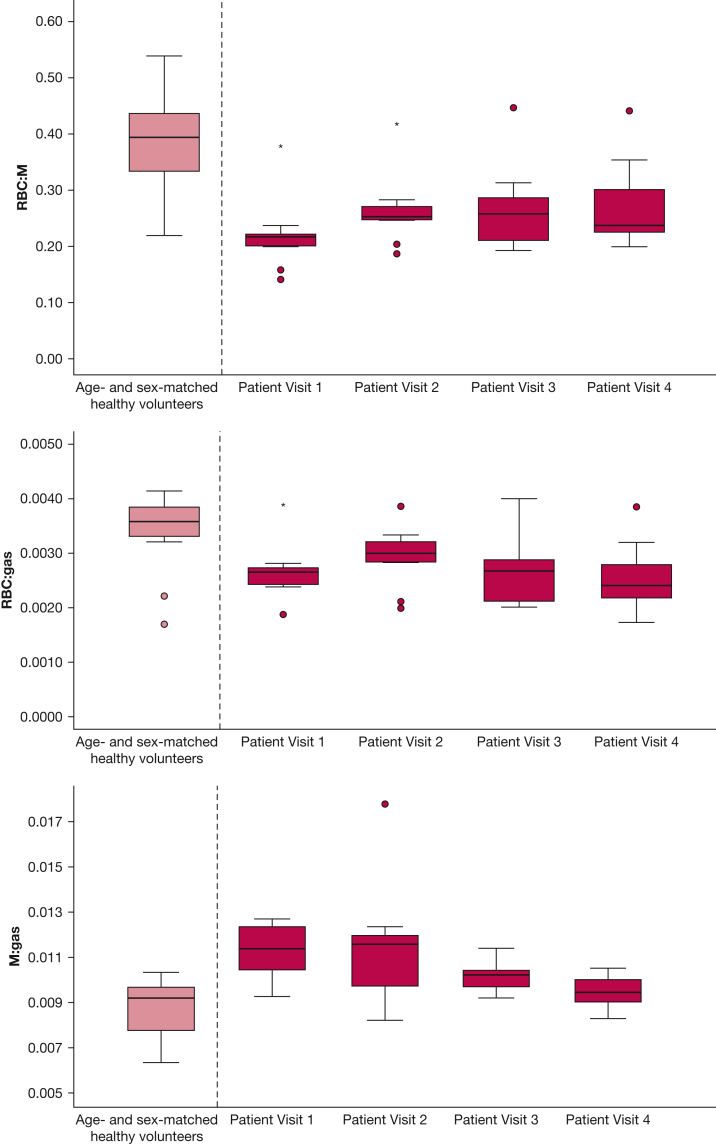
Boxplots of xenon gas transfer ratios from patients at visits 1 to 4 as well as metrics from an age- and sex-matched healthy cohort. Open circles denote data > 1.5 interquartile range; star denotes data > 3 interquartile range. M = membrane.; M:gas = membrane to gas fraction; RBC:gas = RBC to gas fraction; RBC:M = RBC to membrane fraction.

Figure 6 shows boxplots of the RBC:M, RBC:gas, and M:gas for patients at each visit, with reference boxplots of age- and sex-matched control data (control RBC:M median, 0.39 [IQR, 0.13]; RBC:gas median, 0.0034 [IQR, 0.0006]; M:gas median, 0.0088 [IQR, 0.0021]). The number of patients who had RBC:M below the median ± IQR of the age- and sex-matched healthy volunteers was eight of nine at visit 1, seven of nine at visit 2, five of seven at visit 3, and six of eight at visit 4.

**e-Fig 3:**
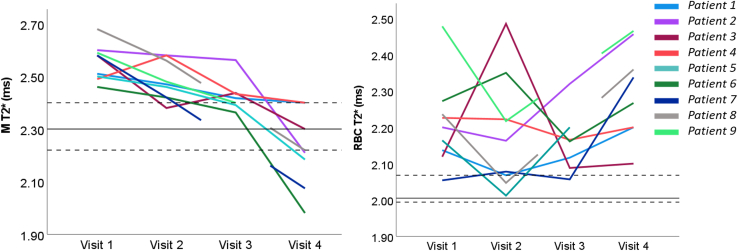


The T_2_∗ of the M and RBCs was calculated. M T_2_∗ showed a significant longitudinal decrease across visits, with lower M T_2_∗ at visit 4 compared with visits 1, 2, and 3 (*P*_adj_ = .023, *P*_adj_ = .023, and *P*_adj_ = .047, respectively) and between visits 1 and 3 (*P*_adj_ = .031) (Table 2). No other significant changes in the T_2_∗ of the RBC or M were seen (e-Fig 3).

### ^1^H MRI

#### Structural Changes

The UTE image of Patient 3 showed abnormal linear parenchymal changes at visit 1, which improved but remained abnormal at visits 2 and 3 and were resolved at visit 4. Patients 2, 6, 7, and 8 displayed air trapping on their UTE image at visit 1, which resolved at visit 2 for patients 6, 7, and 8. Patient 2 continued to have air trapping present at visits 3 and 4. The UTE images of Patients 1, 4, 5, and 9 were normal at all visits (e-Table 2).

#### DCE (Perfusion)

Patient 1 showed a segmental perfusion defect at visit 1 that was resolved at visit 2. No other patients showed any substantial regional perfusion defects. Median pulmonary blood volume and flow increased in all patients (n = 6) at visit 2 compared with visit 1; however, the increase was not statistically significant. For the six patients with DCE MRI at visits 1 and 2, median pulmonary blood volume was 37.8 (11.7-53.5) mL/100 mL at visit 1 and 47.6 (15.0-60.2) mL/100 mL at visit 2, and pulmonary blood flow was 76.9 (19.6-107.2) mL/100 mL/min at visit 1 and 91.1 (30.7-109.5) mL/100 mL/min at visit 2 (Fig 4).

### Pulmonary Function Tests

**Figure 7:**
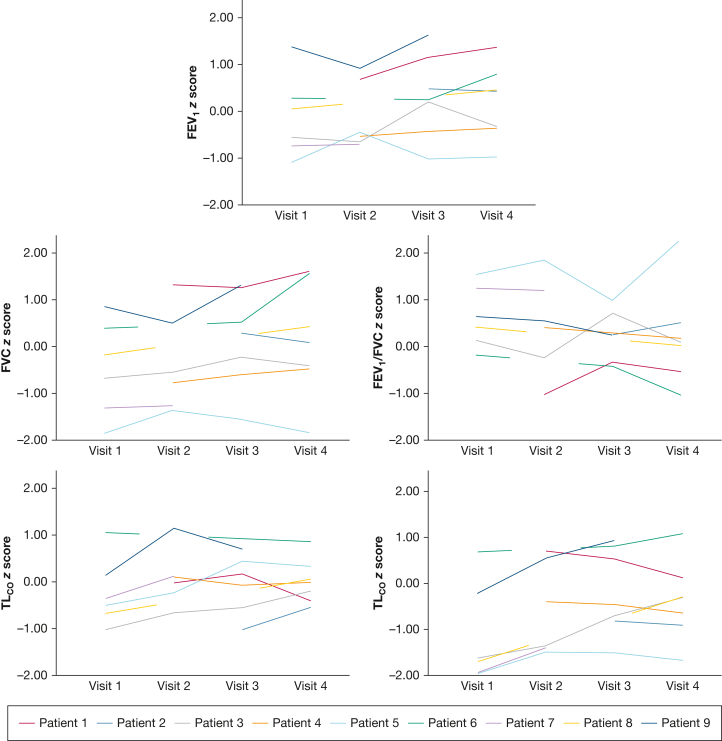
Spaghetti plots of FEV_1_*z* score, FVC *z* score, FEV_1_/FVC *z* score, K_CO_*z* score, and TL_CO_*z* score. K_CO_ = carbon monoxide transfer coefficient; TL_CO_ = transfer factor for carbon monoxide.

Data were available on PFTs for six of nine patients at visit 1, six of nine patients at visit 2, seven of seven patients at visit 3, and seven of eight patients at visit 4; all *z* scores and % predicted data are shown in Figure 7. There was a median of 0 days (mean, 2.8 days; range, 0-23 days) between MRI and PFTs.

Median TL_CO_ *z* score was –1.66 (–1.96 to 0.66) at visit 1, –0.88 (–1.49 to 0.68) at visit 2, –0.47 (–1.51 to 0.90) at visit 3, and –0.31 (–1.67 to 1.05) at visit 4. Three of six patients had an abnormal TL_CO_ *z* score (< 1.64) at visit 1. No patients had an abnormal TL_CO_ *z* score at visit 2 or 3. One patient had an abnormal TL_CO_ *z* score at visit 4.

One patient (Patient 5) had abnormally low FVC at visit 1 and visit 4. No other forced lung volume metrics were abnormal at any visits.

### Linear Mixed-Effect Model of RBC:M

A significant increase in RBC:M was found with increasing pulmonary blood volume, pulmonary blood flow, decreasing VDP, and increasing TL_CO_ *z* score, using data from all 4 visits (Table 3). No statistically significant relationship was found between RBC:M and mean transit time.

**Table 3:** Effect of Pulmonary Blood Volume, Pulmonary Blood Flow, Mean Transit Time, VDP, and TL_CO_*z* Score on RBC:M Tested Using Linear Mixed-Effect Model Analysis With a Random Intercept

Independent Variable	Estimated Coefficient	*P* Value	Lower CI	Upper CI
Pulmonary blood volume (mL/100 mL)	0.0016	.002	0.0007	0.0025
Pulmonary blood flow (mL/100 mL/min)	0.00067	.015	0.00014	0.00120
Mean transit time (s)	0.0082	.076	–0.00093	0.01729
VDP (%)	–0.025	.009	–0.0427	–0.007
TL_CO_*z* score	0.048	< .001	0.027	0.069

M = membrane; RBC:M = RBC to membrane fraction; TL_CO_ = transfer factor for carbon monoxide; VDP = ventilation defect percentage.

## Discussion

This study used a comprehensive ^1^H and ^129^Xe MRI protocol to assess pathophysiologic pulmonary changes in hospitalized patients with COVID-19 for up to 1 year following hospitalization. At 6 weeks following hospitalization, four of nine patients had small ventilation defects, TL_CO_ *z* score was abnormal in three of nine patients, and xenon gas transfer (RBC:M) was outside the median ± IQR of age- and sex-matched healthy subjects in eight of nine patients. At 12 weeks, improvements were seen in lung ventilation and xenon gas transfer. However, there was no longitudinal change in xenon gas transfer between 12 and 52 weeks, and median ^129^Xe gas transfer in these patients remained lower than expected. This indicates that some of the patients with COVID-19 exhibited continued abnormalities in ^129^Xe gas transfer at 12 to 51 weeks following hospitalization, despite normal lung structural imaging and ventilation, with six of eight patients outside the median ± IQR of normal age- and sex-matched patients at 51 weeks.

Several studies have reported reduced gas transfer to the RBCs in patients hospitalized due to COVID-19.^21^, ^22^, ^23^ Because xenon gas transfer depends on both the xenon uptake in the lung tissue and the xenon uptake in the RBCs, a combination of lung perfusion abnormalities and/or alveolar/interstitial endothelial changes may be mechanistically driving the reduced xenon gas transfer seen in patients following COVID-19. Although not directly comparable to the results from those studies due to differences in imaging parameters, our findings are in accordance with the reporting of significantly lower RBC:M values between hospital discharge and 24 weeks postdischarge in previous studies.^20^, ^21^, ^22^ In the current study, the inclusion of data from age- and sex-matched healthy control subjects shows that these changes are not due to age or sex differences between control subjects and patients in this study. RBC:gas and M:gas did not show significant longitudinal change once adjusted for multiple comparisons, implying that the change in RBC:M was a combined effect of changes in both M and RBC. A significant reduction in M T_2_∗ at visit 2 was also found. The physiological mechanisms behind changes in M T_2_∗ are not well established and are discussed further in the Discussion section of e-Appendix 1

We also found that changes in xenon gas transfer increased significantly with increased TL_CO_ *z* score, VDP, and lung perfusion metrics (pulmonary blood volume and pulmonary blood flow). All patients with DCE data available displayed an increase in regional pulmonary blood flow and volume between visits 1 and 2, despite only one having a substantial perfusion defect. This may indicate microvascular improvements at 12 weeks, and that microvascular recovery may be partially driving changes in RBC:M in these patients. In parallel, a concomitant reduction in M signal due to resolution of postinfection endothelial inflammation could contribute to the increase in RBC:M with time.

Although we see global correlations between RBC:M, ventilation, and perfusion, regional heterogeneity in RBC:M did not visually agree with ventilation or perfusion heterogeneity; for example, as shown in Figure 3, Patient 8 has a visually heterogeneous RBC:M map but no visual concordance with pulmonary blood flow heterogeneity and homogeneous ventilation on the similar slices presented. Further work assessing regional distributions seen in the different functional magnetic resonance images available here is warranted to evaluate regional correlations quantitatively.

In this study, most patients (seven of nine) did not report significant breathlessness at visit 2 (12 weeks), despite lower RBC:M than the control reference data. The two patients who reported breathlessness at visit 2 had the two lowest RBC:M values at that visit. Larger studies in symptomatic patients are needed to further investigate links between RBC:M and breathlessness or other post-COVID-19 symptoms. Fully recovered post-COVID-19 control groups will be important in further studies investigating post-COVID-19 breathlessness with these imaging techniques.

Median patient ADC and Lm_D_ were within the age- and sex-matched reference range at visits 1 and 2,^35^ with no significant change at visit 2, indicating that airway dimensions were not increased in these nine patients who had COVID-19 but no signs of interstitial lung damage on structural imaging. This study excluded patients with signs of interstitial lung damage, as previous work has shown that patients with interstitial lung diseases can have reduced xenon gas transfer,^6^ alterations in lung microstructure measured using ^129^Xe MRI,^6^ reductions in lung ventilation,^46^ and reductions in lung perfusion.^47^ Although this means that there is considerable promise for lung MRI to provide longitudinal biomarkers in patients with signs of interstitial lung damage, it also suggests that persistent perfusion, ventilation, gas transfer, and lung microstructure abnormalities may be mechanistically related to the visible tissue changes within a cohort with structural lung abnormalities. Further work using a ^1^H and ^129^Xe protocol in patients with established pulmonary fibrosis due to COVID-19 on CT scan imaging is the subject of an ongoing study (UKILD).^29^

Minor ventilation heterogeneity and defects were present in this cohort shortly following acute illness; these defects improved over time, which is consistent with the findings of Grist et al^21^ and of Li et al.^20^ Overall, the current study and the findings from previous literature suggest it is unlikely that impaired lung ventilation is the primary cause of ongoing symptoms following the acute stage of COVID-19 and that the pathophysiology is not primarily of the airways.

The main limitation of the current study is the limited number of participants, which was largely caused by the challenging nature of recruiting patients for scanning directly following a recent hospitalization due to COVID-19 in the first wave of the pandemic. In addition, not all patients had DCE lung perfusion imaging or PFTs at all examinations (due to aerosolization constraints). The numbers recruited limit correlations with symptoms, activity, and lung function, as well as the statistical tests used to test for change. All ^129^Xe acquisitions were acquired at functional residual capacity plus 1 L, resulting in some variability between patients in the lung inflation state. A final potential source of bias in this study is that five patients who were potentially eligible for the study were excluded due to chest size exceeding the size of the xenon MRI coil.

## Interpretation

This study found that in a cohort of patients who were hospitalized with COVID-19 pneumonia of moderate severity who had normal CT scan/lung structural imaging, ^129^Xe gas transfer improved at 12 weeks but did not return to within a normal range within 1 year following hospitalization. Improvements in ^129^Xe gas transfer were associated with an increased lung perfusion on DCE-MRI and increased TL_CO_ *z* score; therefore, abnormalities in ^129^Xe gas transfer may be a marker of ongoing microvascular abnormalities post-COVID-19.

TL_CO_ *z* score was within a normal range for seven of eight patients with available data at 51 weeks posthospitalization. This indicates that ^129^Xe gas transfer may be a more sensitive measure of gas exchange in this population and that it may be able to identify abnormalities that routine clinical tests overlook.

We believe this to be the first follow-up study of similar patients with such an extensive range of functional lung imaging techniques. Our findings show the sensitivity and complementary nature of functional MRI to follow-up post-COVID-19 lung pathophysiology in a clinical setting.

## Funding/Support

This study was supported by a Medical Research Council grant (MR/M008894/1), a GSK investigator-led grant funding (R/167242-11-1) and a GE Healthcare investigator-led grant funding (R/167303-11-1) to J. M. W. A. A. R. T. was supported by a British Heart Foundation Intermediate Clinical Fellowship (FS/18/13/33281). The study was supported by the NIHR Sheffield Biomedical Research Centre (BRC) / NIHR Sheffield Clinical Research Facility (CRF). Grant funding from the NIHR was received by A. J. S. (AI award: AI_AWARD01706), F. G. (EXPLAIN study: COV-LT2-0049), and K. M. H. for the development of an MRI sequence used in this work (5R01HL136965).

## Financial/Nonfinancial Disclosures

The authors have reported to *CHEST* the following: The following authors have declarations of support from organizations for the submitted work: P. J. C. H. receives grant funding from GSK and Bayer. A. A. R. T. is funded by British Heart Foundation Intermediate Clinical Fellowship and grant funding from Janssen-Cilag Ltd. A. L. receives grant funding from the British Heart Foundation (fellowship award). R. G. J. receives grant funding from AstraZeneca, Biogen, Galecto, GSK, RedX, Pliant, and Genentech. A. J. S. receives grant funding from the National Institute for Health and Care Research (NIHR) (AI award), Wellcome (Innovator award), and Janssen-Cilag Ltd (project grant). J. M. W. receives grant funding from the Medical Research Council, GSK (investigator led research grant), and GE Healthcare. F. G. receives grant or contract funding from Oxford NIHR Biomedical Research Centre, NIHR (HypErpolarised Xenon Magnetic Resonance PuLmonary Imaging in PAtIeNts with Long-COVID - EXPLAIN), POLAREAN Ltd, and GE Healthcare. K. M. J. has received National Institutes of Health grant funding for the development of an MRI sequence used in this work. The following authors declare financial relationships with organizations that might have an interest in the submitted work in the previous 3 years: A. C. is an employee of GSK and is a shareholder in GSK. F. J. W. was an employee of GSK and was a shareholder in GSK at the time of the study. R F. S. is employed by, and a shareholder of, GE Healthcare. A. L. receives funding support from Janssen-Cilag Ltd for meetings/travel. A. A. R. T. receives funding from Janssen-Cilag Ltd for meetings/travel. R. G. J. receives consulting fees from Bristol Myers Squibb, Daewoong, Veracyte, Resolution Therapeutics, RedX, Pliant, and Chiesi. A. J. S. receives consultancy fees from Janssen-Cilag Ltd. R. G. J. receives payment from Chiesi, Roche, patientMpower, AstraZeneca, GSK, and Boehringer Ingelheim for lectures. A J. S. receives payment for presentations from Janssen-Cilag Ltd. F. G. receives payment for attending a research advice meeting from POLAREAN Ltd. R. G. J. is a trustee of Action for Pulmonary Fibrosis. F. G. is the president of the European Society for Thoracic Imaging. None declared (L. C. S., G. J. C., H.-F. C., L. J. S., J. G. R. W., J. E. M., Z. G., T. N., M. P., J. A. E., S. T., S. S., L. G., H. M., J. B., D. A. C., L. A., J. R., M. B., A. M. B., G. N., O. R., M. R. R., R. M., N. J. S., J. T. G., S. R., G. H. M., R. L., P. J. C., J. E. B., P. W., L. W.).

## Supplementary Data

e-Online Data 1

e-Online Data 2

e-Online Data 3
