# Supplementary material for: Longitudinal Lung Function Assessment of Patients Hospitalized With COVID-19 Using 1H and 129Xe Lung MRI
Source: Chest. 2023 Mar 24;164(3):700–16. doi: 10.1016/j.chest.2023.03.024 (PMC10036146; doi:10.1016/j.chest.2023.03.024)
Supplement: e-Online Data 1 [file mmc1.docx]

**Manuscript title:** Longitudinal lung function assessment of patients hospitalised with COVID-19 using ^1^H and ^129^Xe lung MRI

**Supplementary material – figure legends**

**E-Figure 1**: Lung ventilation images for patients 1 and 3, who showed small peripheral lung ventilation defects at visit 1 which partially resolve at visits 2 and 3. White arrows indicate defect areas. Mean VDP at each visit is shown. VDP: ventilation defect percentage.

**E-Figure 2:** Box plot of LmD from patients at visits 1-4, alongside age and sex matched healthy volunteer data. >1.5 IQR, ★ denotes data >3 IQR.

**E-Figure 3**: Spaghetti plots of T_2_* of the ^129^Xe signal observed in the tissue and blood. M T_2_* was significantly lower at visit 3 compared to visit 1, and visits 1, 2 and 3 compared to visit 4. Solid and dotted lines show age and sex matched healthy volunteer median and interquartile range, respectively. RBC: red blood cell. M: membrane.
