# Supplementary material for: Longitudinal Lung Function Assessment of Patients Hospitalized With COVID-19 Using 1H and 129Xe Lung MRI
Source: Chest. 2023 Mar 24;164(3):700–16. doi: 10.1016/j.chest.2023.03.024 (PMC10036146; doi:10.1016/j.chest.2023.03.024)
Supplement: e-Online Data 2 [file mmc2.docx]

**Manuscript title:** Longitudinal lung function assessment of patients hospitalised with COVID-19 using ^1^H and ^129^Xe lung MRI

**Supplementary material – tables**

**E-table 1**: typical imaging parameters used for MRI acquisition

|  | ^129^Xe ventilation | ^129^Xe DW-MRI | Dissolved phase ^129^Xe | ^1^H UTE | ^1^H VFA | ^1^H DCE |
| --- | --- | --- | --- | --- | --- | --- |
| Sequence | 3D bSSFP sequence | 3D SPGR multiple b-value with elliptical-centric phase encoding | 4-echo flyback 3D radial technique | 3D SPGR radial sequence | 3D SPGR | 3D SPGR |
| Lung coverage | 3D volumetric whole lung coverage | 3D volumetric whole lung coverage | 3D volumetric whole lung coverage | 3D volumetric whole lung coverage | 3D volumetric whole lung coverage | 3D volumetric whole lung coverage |
| Acquisition matrix | 100x100 | 64 × 52 | 32x32x32 | 256 x 256 x 256 | 200x80 | 200x80 |
| Field of view (cm^2^) | 48 | 48 | 40 | 35 | 48 | 48 |
| Slice thickness (mm) | 10 | 15 | 0.55 | 1.37 | 4 | 10 |
| TE (ms) | 2.17 | 14.0 |  | 0.08 | 0.9 | 0.69 |
| TR (ms) | 6.52 | 17.4 | 15 | 2.9 | 2.85 | 2.08 |
| FA (°) | 10 | 3 | 22 | 4 | 2°, 4°, 10°, 30° | 30° |
| Temporal acquisitions | - | - | - | - | - | 48 |
| Temporal resolution (s) | - | - | - | - | - | ~0.5 |
| SENSE factor | - | - | - | - | - | 2 |
| Xenon dose | 500ml | 550ml | 1000ml | - | - | - |
| Bandwidth (Hz/pixel) | 62.97 | 54.22 | 31.25 | 1953.10 | 488.28 | 976.56 |
| Respiratory state | FRC + 1L max bag of gas (^129^Xe and N_2_ mixture) | FRC + 1L max bag of gas (^129^Xe and N_2_ mixture) | FRC + 1L max bag of gas (^129^Xe) | Expiration, prospectively gated free breathing | Expiration breath hold | Expiration breath hold followed by shallow breathing |
| Additional parameters |  | b = 0, 12, 20, 30 s/cm^2^  diffusion time (Δ) = 8.5 ms | ΔTE = 0.7 |  |  |  |

**Supplementary table 2:** UTE radiological assessment.

| **UTE radiological assessment** | | | | |
| --- | --- | --- | --- | --- |
|  | **Visit 1** | **Visit 2** | **Visit 3** | **Visit 4** |
| **Patient 1** | Normal | Normal | Normal | Normal |
| **Patient 2** | Air trapping | Air trapping | Air trapping | Very minor air trapping |
| **Patient 3** | Abnormal linear parenchymal changes and air trapping. | Parenchymal changes are present but improved. | Parenchymal changes are present but further improved. | Normal |
| **Patient 4** | Normal | Normal | Normal | Normal |
| **Patient 5** | Normal | Normal | Normal | Normal |
| **Patient 6** | Air trapping | Normal | Normal | Normal |
| **Patient 7** | Air trapping | Normal | - | Normal |
| **Patient 8** | Air trapping | Normal | - | Normal |
| **Patient 9** | Normal | Normal | Normal | - |
