# Supplementary material for: Longitudinal Lung Function Assessment of Patients Hospitalized With COVID-19 Using 1H and 129Xe Lung MRI
Source: Chest. 2023 Mar 24;164(3):700–16. doi: 10.1016/j.chest.2023.03.024 (PMC10036146; doi:10.1016/j.chest.2023.03.024)
Supplement: e-Online Data 3 [file mmc3.docx]

**Manuscript title:** Longitudinal lung function assessment of patients hospitalised with COVID-19 using ^1^H and ^129^Xe lung MRI

**Supplementary text**

**Methods:**

***Dissolved phase xenon acquisition and analysis***

The spectroscopic imaging method for the first visit of subjects 1 and 2 followed the 4-echo flyback 3D radial technique[1]. For the remaining visits and all other subjects, an updated version of the imaging method was implemented. The main improvements being: (i) the implementation of a frequency-tailored RF excitation pulse that excites the gas phase with 1% of the flip angle on tissue and blood, to allow a more efficient sampling time by removing the interleaved RF excitation between gas and dissolved phase and increase the number of radial projections from 332 to 934, which therefore decreased the radial undersampling; (ii) the TR was shortened to 15 ms and the flip angle reduced from 40 to 22 degrees to match the imaging parameters recommended by the ^129^Xe MRI clinical trials consortium[13]; (iii) the calibration spectrum required to estimate the frequency positions and transverse relaxation times (T_2_^*^) of each resonance was inserted at the start of the imaging sequence. These estimates were used as prior information to improve the chemical shift reconstruction and were derived by performing a triple Lorentzian fit of the data in the frequency domain[14]. This latter addition removed the use of a separate calibration dose and sequence in the imaging protocol. The remaining imaging parameters (FOV, matrix size, BW, echo times) stayed the same and are shown in the supplementary table 1.

A correction factor for the visit 1 of subjects 1 and 2 RBC:M ratio was applied to account for the different TR and flip angle between the two implementations of the dissolved phase imaging technique. The correction factor was estimated using images acquired using both techniques on 6 healthy volunteers. RBC:Gas and M:Gas ratio were discarded for these patients (n=2), as a reliable correction factor was not found.

***Linear binning methodology for lung ventilation imaging***

A linear binning approach[2] based on [3] was used to compute a 4 level grading of the ventilation images as follows: (1) pre-computed estimations of the lung cavity and major airways[4] were manually edited; (2) the N4 bias field correction filter[19] was applied to the ventilation images, where the ventilated-region mask was supplied by an in-house-developed deep-learning tool; (3) finally, normalisation by the mean signal inside the lung cavity and linear binning were performed with the lower bins being the defect and low ventilation regions. Coefficient of variation (CV) of ventilation images was calculated from the 3D ventilated volume as a measure of ventilation heterogeneity[5].

***UTE imaging***

A correction for receiver coil signal non-uniformity (PURE) was performed retrospectively on all images using GE Orchestra software.

***Lung perfusion imaging***

Co-registration between the variable flip angle images and dynamic lung perfusion acquisition was performed using ANTs image registration toolkit[6] to allow voxelwise correction for lung T_1_ and proton density values.

**Discussion:**

A significant reduction in M T_2_^*^ at visit 3 and compared to visit 1, and at visit 4 compared to visits 1, 2 and 3 (see Table 2). The physiological mechanisms behind changes in M T_2_^*^ are not well established. An increased M T_2_^*^ at visit 1 may be due to residual inflammation increasing the thickness of interstitial tissue, potentially resulting in a decrease in microscopic field inhomogeneity effects in the interstitium. However, further work is warranted to determine this relationship.

**Supplement References**

1. Collier, G.J., et al., *Dissolved (129) Xe lung MRI with four-echo 3D radial spectroscopic imaging: Quantification of regional gas transfer in idiopathic pulmonary fibrosis.* Magn Reson Med, 2021. **85**(5): p. 2622-2633.

2. Collier, G., *Linear binning maps for image analysis of pulmonary ventilation with hyperpolarized gas MRI: transferability and clinical applications.* , in *International Society for Magnetic Resonance in Medicine*. 2018: Paris.

3. He, M., et al., *Using Hyperpolarized (129)Xe MRI to Quantify the Pulmonary Ventilation Distribution.* Acad Radiol, 2016. **23**(12): p. 1521-1531.

4. Hughes, P.J.C., et al., *Spatial fuzzy c-means thresholding for semiautomated calculation of percentage lung ventilated volume from hyperpolarized gas and (1) H MRI.* J Magn Reson Imaging, 2018. **47**(3): p. 640-646.

5. Hughes, P.J.C., et al., *Assessment of the influence of lung inflation state on the quantitative parameters derived from hyperpolarized gas lung ventilation MRI in healthy volunteers.* J Appl Physiol (1985), 2019. **126**(1): p. 183-192.

6. Avants, B.B., et al., *A reproducible evaluation of ANTs similarity metric performance in brain image registration.* Neuroimage, 2011. **54**(3): p. 2033-44.
